# Médico-Pesquisador, Prática Médica e Pesquisa: A Importância do Médico-Pesquisador na Medicina Atual

**DOI:** 10.36660/abc.20220099

**Published:** 2022-11-01

**Authors:** Protásio Lemos da Luz

**Affiliations:** 1 Hospital das Clínicas da Faculdade de Medicina da Universidade de São Paulo Instituto do Coração São Paulo SP Brasil Instituto do Coração do Hospital das Clínicas da Faculdade de Medicina da Universidade de São Paulo, São Paulo, SP – Brasil

**Keywords:** Médicos, Pesquisadores, Faculdades de Medicina, Educação Médica, Pesquisa Médica

*“Não se aprende, Senhor, na fantasia*

*Sonhando, imaginando ou estudando*

*Senão vendo, tratando e pelejando”*

**Camões, sobre a arte militar, em *Os Lusíadas*.**

**O mesmo se aplica à arte médica.**

O ensino e a prática médica brasileiros sofrem de inúmeras limitações, entre as quais destaca-se a deficiente formação científica. Ultimamente surgiu a figura do médico-pesquisador como elemento capaz de suprir, em parte, tal deficiência. Aqui procuro avaliar seu papel.

A medicina tradicional ocupa-se da aplicação do conhecimento médico. Portanto, lida com as características gerais das doenças, isto é, sintomas, evolução, comprometimento fisiopatológico de órgãos e sistemas, etiologia, métodos diagnósticos e terapêutica.

Requer estruturas hospitalares, ambulatoriais e laboratoriais clássicas que permitam fazer testes diagnósticos como imagens e perfil bioquímico. No exercício da clínica não se permite experimentação. O exercício da profissão médica baseia-se na aplicação judiciosa do conhecimento atual.

Uma das grandes dificuldades nesse processo é que as doenças não ocorrem como puros fenômenos biológicos, bioquímicos ou físicos. Ao contrário, como as doenças ocorrem nas pessoas, suas manifestações são dependentes da individualidade de cada um. As escolas médicas procuram mostrar aos alunos o estado atual das enfermidades e como elas são investigadas e tratadas. Ocorre que as verdades biomédicas mudam com a evolução do conhecimento, e isto esta ocorrendo atualmente com grande velocidade dado a evolução das pesquisas e do conhecimento médico. Um aspecto essencial do ensino médico é o conceito de que se deve não apenas ensinar o que se sabe hoje, mas ensinar o aluno a pensar criticamente para poder julgar as novidades publicadas e aplicar apenas conhecimentos bem estabelecidos com base em pesquisas de alto nível. Evidentemente, se o professor, ele mesmo, não vivenciar este conceito, não poderá transmiti-lo aos alunos. Portanto, a qualidade da prática médica depende não apenas do ensino, mas também das estruturas do sistema de saúde como um todo.

A qualidade do ensino médico atual no Brasil é inadequada. Por exemplo, o exame do Conselho Regional de Medicina do Estado de São Paulo (CREMESP), que é feito voluntariamente por alunos ao final do curso médico, mostra um índice de aprovação de apenas 50%.^1^ Ora, o CREMESP está restrito ao estado de São Paulo, que tem justamente algumas das melhores escolas médicas do país. Pode-se imaginar qual seria o resultado de exame semelhante se fosse aplicado a todas as escolas médicas do país, muitas das quais não têm estrutura técnica nem corpo docente qualificado. A criação desordenada de escolas médicas nos últimos anos é causa de imensa preocupação entre os profissionais dedicados ao ensino médico. Evidentemente, pode-se esperar que grande número de médicos pouco qualificados passe a exercer a profissão nos próximos anos. Isso representa uma ameaça ao atendimento médico da população em geral.

Para complicar, o número de hospitais de ensino é notoriamente insuficiente. Assim, das 353 escolas médicas apenas 69 (19,5%) têm hospitais de ensino.^2,3^ Além disso, os hospitais de ensino, em geral, não possuem estruturas para treinamento em pesquisa.

Em resumo, pode-se concluir que o ensino médico no Brasil, em geral, é de baixa qualidade. Por extensão a prática médica é também de baixo nível. Como não há nenhum controle externo cada médico exerce a profissão segundo suas próprias convicções. Daí resultam aplicações “*off-label*” e inúmeros procedimentos questionáveis, tais como uso excessivo de exames laboratoriais e de imagens e prescrições inadequadas de medicamentos sem comprovação de eficácia. Isso, obviamente, onera o sistema de saúde sem vantagens para os pacientes. É neste contexto que se insere o médico-pesquisador, tanto de formação em ciência básica quanto clínica.

Para começar, a formação do pesquisador requer uma formulação especial. O pesquisador precisa entender de metodologia científica *lato sensu*. Especificamente, precisa compreender a importância dos modelos experimentais, sejam de bancada, experimentação animal ou clínicos. Registros baseados em prontuários médicos eletrônicos, com grande número de pacientes, são hoje amplamente utilizados como complementação aos estudos randomizados, e representam o chamado “mundo real”.^4^ O pesquisador precisa entender princípios básicos de estatística, que hoje se tornou uma disciplina por si só, tornando-se indispensável. Precisa compreender bem a questão de vieses diversos, como os de seleção de amostras e a presença de fatores de confusão, sobretudo em estudos observacionais. É obrigatório o entendimento da adequação das amostras e modelos para a resposta de perguntas específicas. Um exemplo muito ilustrativo é o de Eric Kandel,^5^ que no seu magnífico livro *Em busca da memória* relata que passou quatorze meses em Paris, somente estudando as características da lesma marinha Aplysia, que se revelou o modelo ideal para seus estudos de memória. Ele ganhou o Prêmio Nobel de Medicina e Fisiologia em 2000, pelas suas descobertas sobre a memória. Além disso, tecnologias modernas como biologia molecular, genética e epigenética, randomização mendeliana, várias técnicas de imagem de órgãos, tecidos e estruturas intracelulares como moléculas e sinalização paracelular e intracelular, acrescentaram recentemente instrumentos poderosos para a criação de conhecimento.

Diferentemente do que se observa na prática clínica, os resultados de pesquisas são em geral de longo prazo, podendo consumir anos ou até a vida inteira do investigador. Em resumo, a formação do pesquisador é um aprendizado em serviço, que envolve muitas disciplinas, e cuja finalidade precípua é a busca da verdade e a criação de novos conhecimentos.

A relevância do médico-pesquisador fica clara dentro do escopo de associar clínica e pesquisa; alguém que tenha visão conjunta, integrada dos grandes problemas médicos. Isso tem a ver com eficiência, relevância, inovação e a noção de custo/benefício. No entanto, é preciso conhecimento mínimo da parte do médico-pesquisador sobre aspectos essenciais da medicina de hoje: genética e epigenética, biologia molecular, biologia vascular, sistemas de sinalização celulares e intracelulares, sistemas redox entre outros. Especificamente cabe a este: a) analisar criticamente os resultados de pesquisas sejam experimentais ou aplicadas, com o objetivo de verificar se os procedimentos empregados são de alta qualidade e, portanto, confiáveis. Exemplos recentes de publicações sem base científica sólida, são os múltiplos estudos relativos ao tratamento medicamentoso da Covid-19; várias propostas terapêuticas se revelaram totalmente inócuas ou até prejudiciais, causando sérios transtornos no tratamento dessa pandemia.^6^ Parte desse problema se deve às publicações “pré-print”, sem aval de uma revisão por pares. Casos similares são os de vitamina E,^7^ reposição hormonal^8^ e antioxidantes.^9^ cujas hipóteses de benefícios não se comprovaram; b) interpretar e divulgar os achados de investigações para o clínico, já salientando os prós e contras desses achados; c) gerar protocolos relevantes que possam ser desenvolvidos no nosso meio; d) criar os meios adequados para o desenvolvimento de pesquisas, de novas tecnologias ou a implementação de desenvolvimentos recentes. Esta talvez seja a missão mais importante do médico-pesquisador. Hospitais, com exceção dos de ensino, são normalmente preparados para atendimento médico. Portanto não têm estruturas próprias para pesquisas. Habitualmente gestores administrativos estão mais preocupados com finanças e eficiência do complexo hospitalar. No entanto, conceitos modernos indicam que ao praticar investigações clínicas, a qualidade do atendimento médico melhora. Assim, associar atendimento e pesquisas é uma maneira de aprender com a própria experiência e maximizar os efeitos do trabalho. É uma maneira de se evitar erros repetitivos, pela avaliação crítica e constante dos procedimentos de uma instituição. Esta é uma tendência da medicina moderna.

Estas funções do médico-pesquisador, são essenciais na medicina translacional,^10^ compreendendo desde a divulgação de achados básicos, passando pela elucidação de mecanismos fisiopatológicos e síndromes clínicas e sobretudo encurtando o período entre descobertas de ciências básicas e sua aplicação clínica. Portanto, a atuação do médico-pesquisador é essencial para o exercício qualificado da profissão médica, bem como para o desenvolvimento científico do país.

Em conclusão, o médico-pesquisador pode exercer papel fundamental no aperfeiçoamento do ensino e da prática médica. Seu conhecimento do método científico pode contribuir para o exercício de uma medicina criteriosa, baseada em investigações realmente qualificadas.

Felizmente, temos observado nos últimos anos, no Brasil um número crescente de médicos-pesquisadores. Porém, estes números são ainda insuficientes. Portanto, devemos incentivar iniciações científicas e cursos de pós-graduação qualificados no Brasil e no exterior e sobretudo adequar escolas médicas para não apenas exercer seu papel tradicional mas também criar estruturas de pesquisa.
