# Supplementary material for: Médico-Pesquisador, Prática Médica e Pesquisa: A Importância do Médico-Pesquisador na Medicina Atual
Source: Arq Bras Cardiol. 2022 Nov 1;119(5):801–3. [Article in Portuguese] doi: 10.36660/abc.20220099 (PMC9750216; doi:10.36660/abc.20220099)
Supplement: Translation

# Translation: Physician-Researcher, Medical Practice and Research: The Importance of the Physician-Researcher in Current Medicine

**Authors:** Protásio Lemos da Luz

**Affiliations:** 1 São Paulo SP Brazil Instituto do Coração do Hospital das Clínicas da Faculdade de Medicina da Universidade de São Paulo, São Paulo, SP – Brazil

**Keywords:** Physicians, Research Personnel, Schools, Medical, Education, Medical, Biomedical Research

*“One does not learn, Sir, by fantasy*

*Dreaming, imagining, or studying*

*But rather by seeing, treating, and striving”*

**Luís de Camões, on military arts, in *The Lusiads*.**

**The same applies to the art of medicine**

Medical education and practice in Brazil suffer from countless limitations, among which lacking scientific training stands out. Recently, the physician-researcher has emerged as a figure capable of partially correcting this gap. In this paper, I will seek to evaluate the physician-researcher's role.

Conventional medicine is dedicated to the application of medical knowledge. Therefore, it deals with the general characteristics of diseases, namely, symptoms, evolution, pathophysiological impairment of organs and systems, etiology, diagnostic and therapeutic methods.

This requires conventional hospital, outpatient, and laboratory structures that make it possible to conduct diagnostic tests, such as imaging and biochemical profile. Within clinical practice, experimenting is not permitted. Practice of the medical profession is based on the judicious application of current knowledge.

One of the main difficulties in this process is that diseases do not occur as purely biological, biochemical, or physical phenomena. On the contrary, because diseases occur in people, their manifestations are dependent on the individuality of each person. Medical schools seek to show students the current state of illnesses and how they are investigated and treated. It so happens that biomedical truths change as knowledge evolves, and this is currently taking place at an amazing speed, given the evolution of research and medical knowledge. An essential aspect of medical education is the concept that we must not only teach what is known today, but also teach students to think critically in order to be able to judge published reports and to apply only well-established knowledge that is based on high-level research. Evidently, if educators themselves do not incorporate this concept, they will not be able to transmit it to students. Therefore, the quality of medical practice depends not only on teaching, but also on the structures of the health system as a whole.

The quality of medical education in Brazil is currently inadequate. For example, the Regional Council of Medicine of the State of São Paulo (CREMESP) exam, which is taken voluntarily by students at the end of their medical course, shows an approval rate of only 50%.^1^ CREMESP is limited to the state of São Paulo, which has some of the best medical schools in Brazil. It is possible to imagine what the results of a similar exam would be, were it applied to all Brazilian medical schools, many of which do not possess technical structures or qualified teaching staff. The unprecedent opening of new medical schools in the past years has been a cause of immense concern among professionals dedicated to medical education. Evidently, it is possible to expect that a large number of underqualified doctors will begin practicing the profession in the coming years. This poses a threat to the medical care of the general population.

To complicate matters further, the number of teaching hospitals is notoriously insufficient. Thus, of the 353 medical schools, only 69 (19.5%) have teaching hospitals.^2,3^ Moreover, teaching hospitals, in general, do not have structures for research training.

To summarize, it is reasonable to conclude that medical education in Brazil is, in general, of low quality. By extension, medical practice is also low-level. As there is no external control, each doctor practices the profession according to their own convictions. This results in “off-label” applications and numerous questionable procedures, such as excessive use of laboratory and imaging tests and inappropriate medication prescriptions with no proof of efficacy. This obviously overloads the health system, without any benefits for patients. It is in this context that physician-researchers are positioned, regarding both training in basic science and clinical practice.

To begin with, a researcher's training requires a special formulation. Researchers need to understand *lato sensu* scientific methodology. Specifically, they need to understand the importance of experimental models, whether benchside, animal, or clinical. Registries based on electronic medical records, with large numbers of patients, are now widely used to complement randomized studies, and they represent the so-called “real world”.^4^ Researchers need to understand the basic principles of statistics, which has become a mandatory discipline on its own. They require a deep understanding of the issue of different biases, such as sample selection and the presence of confounding factors, especially in observational studies. It is mandatory to understand the adequacy of samples and models to answer specific questions. A very illustrative example is that of Eric Kandel,^5^ who in his magnificent book *In Search of Memory* reports that he spent fourteen months in Paris, exclusively studying the characteristics of the Aplysia sea slug, which proved to be the ideal model for his studies of memory. He received the Nobel Prize in Medicine or Physiology in 2000 for his discoveries on memory. In addition, modern technologies, such as molecular biology, genetics and epigenetics, Mendelian randomization, as well as various imaging techniques for organs, tissues, and intracellular structures such as molecules and paracellular and intracellular signaling have recently added powerful tools for knowledge creation.

Differently from clinical practice, research results are generally long-term, and they may take years or even a lifetime. A researcher's training is an apprenticeship in service, which involves many disciplines, whose foremost purpose is the search for the truth and the creation of new knowledge.

The physician-researcher's relevance is evident within the scope of associating clinical practice and research to create a joint, integrated view of major medical problems. This is related to efficiency, relevance, innovation, and the notion of cost/benefit. However, a certain amount of knowledge is required on the part of the physician-researcher on essential aspects of contemporary medicine: genetics and epigenetics, molecular biology, vascular biology, cellular and intracellular signaling systems, redox systems, among others. They are specifically responsible for: a) critically analyzing the results of research, whether experimental or applied, with the objective of verifying that the procedures employed are of high quality and, therefore, reliable. Recent examples of publications without a solid scientific basis are the multiple studies related to medical treatment of Covid-19; several therapeutic proposals turned out to be totally innocuous or even harmful, causing serious disorder in the treatment of this pandemic.^6^ Part of this problem is due to “pre-print” publications, which are not backed by peer review. Similar cases are those of vitamin E,^7^ hormone replacement,^8^ and antioxidants,^9^ whose hypothetical benefits have not been proven; b) interpreting and disseminating the findings of investigations to clinicians, highlighting the pros and cons of these findings; c) generating relevant protocols that can be developed in our setting; d) creating adequate means for the development of research and new technology or for the implementation of recent developments. This is perhaps the most important mission of physician-researchers. Hospitals, with the exception of teaching hospitals, are normally prepared for medical care; therefore, they do not have their own research structures. Administrative managers are usually more concerned with the finances and efficiency of the hospital complex. However, modern concepts indicate that, when they practice clinical investigations, the quality of medical care improves. Accordingly, associating care and research is a means of learning from their own experience and maximizing the effects of their work. It is a way to avoid repetitive errors, through constant and critical evaluation of an institution's procedures. This is a trend in modern medicine.

These functions of the physician-researcher are essential in translational medicine,^10^ ranging from the dissemination of basic findings to the elucidation of pathophysiological mechanisms and clinical syndromes and, above all, shortening the period between discoveries of basic sciences and their clinical application. Therefore, the role of the physician-researcher is essential to qualified practice of the medical profession, as well as to the scientific development of the country.

In conclusion, physician-researchers can play a fundamental role in improving teaching and medical practice. Their knowledge of the scientific method can contribute to judicious medical practice, based on truly qualified investigations.

Fortunately, in recent years, we have observed an increasing number of physician-researchers in Brazil. However, these numbers are still insufficient. Therefore, we must encourage scientific initiation and qualified postgraduate courses in Brazil and abroad and, above all, adapt medical schools not only to exercise their traditional role, but also to create research structures.
